# A pilot study of user satisfaction and perceived helpfulness of the Swedish version of the mobile app PTSD Coach

**DOI:** 10.1080/20008198.2018.1472990

**Published:** 2018-05-17

**Authors:** Martin Cernvall, Josefin Sveen, Kerstin Bergh Johannesson, Filip Arnberg

**Affiliations:** Psychiatry, Department of Neuroscience, Uppsala University, Uppsala, Sweden

**Keywords:** Posttraumatic stress, PTSD Coach, mobile phone intervention, mobile apps, smartphones, estrés, postraumático, Entrenador TEPT, intervención por teléfono celular, aplicaciones de teléfono celular, smartphones, 创伤后应激, PTSD Coach, 手机干预, 移动应用, 智能手机, • Participants found the Swedish version of the PTSD Coach slightly to moderately helpful.• There were nominal but statistically non-significant reductions of symptoms of PTSD and depression with medium effect sizes.• Participants had suggestions for improvement of the app.

## Abstract

**Background**: There is a need for accessible interventions in the aftermath of traumatic events with documented efficacy for preventing or reducing negative mental health consequences. The PTSD Coach is a mobile app that has shown to be effective in reducing symptoms of posttraumatic stress (PTSS).

**Objective**: The purpose of the current study was to evaluate the user satisfaction, perceived helpfulness and potential reductions of PTSS and symptoms of depression among participants using the Swedish version of the PTSD Coach.

**Method**: This was an uncontrolled pre-test post-test open trial including participants recruited from the community via advertisement and from an ongoing observational study who had experienced a potentially traumatic event in the last five years. Participants had access to the Swedish PTSD Coach app for four weeks.

**Results**: Eleven participants (mean age = 38.6, female = 8) completed the study. Nine of the participants met criteria for full or partial PTSD. Results from the PTSD Coach Survey indicated that participants found the app slightly to moderately helpful and were slightly to moderately satisfied with the app. Nominal but not statistically significant reductions of medium effect sizes in PTSS (PCL-5) and depression (PHQ-9) from pre- to post-assessment were found. In interviews, participants indicated that they found elements such as learning about PTSD, breathing exercises and monitoring symptoms helpful in managing symptoms. However, several participants indicated that they had not used the app as much as they had intended to. Participants also had suggestions for improvements such as enhanced app structure and better guidance regarding how to use the app.

**Conclusions**: The perceived helpfulness and user satisfaction were slightly lower compared to research on the original version of the app. Experiences from the study are discussed and a future controlled study of the Swedish version of the PTSD Coach is suggested.

## Abstract (es)

**Antecedentes**: Existe una necesidad por intervenciones accesibles en el periodo subsiguiente a los eventos traumáticos que presenten una eficacia documentada en prevenir o reducir las consecuencias negativas de salud mental. El entrenador TEPT (PTSD Coach en sus siglas en inglés) es una aplicación de teléfono celular que ha mostrado ser efectiva en reducir los síntomas de estrés postraumático (SEPT).

**Objetivo**: El propósito del presente estudio fue evaluar la satisfacción del usuario, la utilidad percibida, y las potenciales reducciones de los SEPT y síntomas de depresión entre los participantes de una versión sueca del Entrenador TEPT.

**Método**: Este fue un ensayo abierto no-controlado de evaluación pre y post-intervención, incluyendo participantes que fueron reclutados desde la comunidad usando anuncios y desde un estudio observacional en proceso con quienes han experimentado un evento potencialmente traumático en los últimos cinco años. Los participantes tuvieron acceso a la aplicación móvil Entrenador TEPT sueca durante 4 semanas.

**Resultados**: 11 participantes (edad media = 38.6, mujeres = 8) completaron el estudio. Nueve de los participantes cumplieron los criterios completa o parcialmente para TEPT. Los resultados de la Encuesta de Entrenador TEPT indicó que los participantes encontraron que la aplicación móvil fue desde ligera a moderadamente útil y estuvieron desde ligera a moderadamente satisfechos con la aplicación. Reducciones significativas nominales, pero no estadísticamente significativas, y de efecto de tamaño mediano en TEPT (PCL-5) y depresión (PHQ-9) fueron encontradas desde pre a post-evaluación. En entrevistas, los participantes indicaron que encontraron que elementos tales como aprender sobre TEPT, ejercicios de respiración, y monitorear síntomas; fueron útiles en manejar los síntomas. Sin embargo, varios participantes indicaron que ellos no habían usado la aplicación tanto como ellos habían intentado hacerlo. Los participantes también tuvieron algunas sugerencias para realizar mejoras, tales como una estructura mejorada de la aplicación y mejor guía en cuanto a cómo usar la aplicación.

**Conclusiones**: La utilidad percibida y la satisfacción del usuario fueron levemente más bajas en comparación a la investigación de la versión original de la aplicación. Las experiencias a partir de este estudio son discutidas, y un estudio controlado futuro de la versión sueca del Entrenador TEPT es sugerido.

## Abstract (zh)

背景：在创伤事件发生后，需要采取易于接受的干预措施，并提供有效的预防或减少心理健康负面后果的措施。 PTSD Coach是一款移动应用程序，可以有效减少创伤后应激症状（PTSS）。

目的：本研究的目的是评估使用瑞典版PTSD Coach的参与者的用户满意度、感知到的有用性，和PTSS和抑郁症状的减少。

方法：这是一项非控制的前后测公开试验，通过广告招募，以及通过一项正在进行的观察性研究中从从社区招募过去五年中曾经历过潜在创伤事件的的参与者。 被试可以在4周内使用瑞典PTSD Coach应用程序。

结果：11名参与者（平均年龄= 38.6，女性= 8）完成了研究。 九名被试符合全部或部分PTSD的标准。 PTSD Coach 问卷的结果表明，被试发现该应用程序有略微到中等的帮助，并且对应用程序略微或中等满意。 发现PTSS（PCL-5）和抑郁症（PHQ-9）的减少有中等大小效应，但不是统计学上显著。 在访谈中，被试表示他们发现了诸如了解PTSD，呼吸练习和监测症状等元素有助于治疗。 然而，一些参与者表示他们没有按照本来的计划使用程序。 被试还提出了改进建议，如增强的应用程序结构和有关如何使用应用程序的更好指导。

结论：感知到的有用性和用户满意度比原始版本的应用程序的研究略低。 文章讨论了研究中的经验，并建议未来对瑞典版PTSD Coach进行对照研究。

关键词：创伤后应激; PTSD Coach; 手机干预; 移动应用; 智能手机

## 1. Introduction

A majority of individuals will experience a potentially traumatic event during their lifetime (Frans, Rimmö, Aberg, & Fredrikson, 2005). For some individuals, these experiences are associated with the development of mental health problems such as depression (Mandelli, Petrelli, & Serretti, 2015), substance abuse and stress-related disorders such as posttraumatic stress disorder (PTSD; Arnberg, Bergh Johannesson, & Michel, 2013; Arnberg et al., 2015). However, many individuals experiencing mental health problems after overwhelming events do not receive evidence-based support or treatment (Witteveen et al., 2012), or do not receive treatment until many years after the onset of their problems (Goldstein et al., 2016). There is a need for accessible interventions in the aftermath of traumatic events with documented efficacy for preventing or reducing negative mental health consequences after serious events.

In Sweden, 85% of the population had a smartphone in 2017 (The Internet Foundation in Sweden, 2017). These devices can be used to access self-help interventions aiming to improve both physical and mental health. Currently, there are a plethora of apps available for smartphone users that assert that they help people with their physical or mental health. Although there has been a rapid proliferation of psychological interventions delivered via the Internet for a range of mental health disorders (Andersson & Titov, 2014; Arnberg, Linton, Hultcrantz, Heintz, & Jonsson, 2014), most of these apps lack empirical support, i.e. their efficacy is unknown, and it is therefore important to evaluate mobile apps in scientific studies with rigorous designs (Olff, 2015). In addition, before conducting rigorous studies of novel psychosocial interventions, such as a mobile app, it is important to conduct more basic work evaluating development and feasibility of the intervention (Anderson, 2008).

The PTSD Coach is a mobile app developed by the National Center for PTSD-Dissemination and Training Division, Veterans Administration (VA), by means of participatory research, where focus groups with PTSD patients were used to generate input on features and design (Kuhn et al., 2014). The app was designed to be used either as a stand-alone tool providing psychoeducation and self-management, or to supplement varying levels of care provided by a professional. The app provides psychoeducational information about PTSD and strategies for coping with PTSD symptoms. Versions of the app in English have been publicly available on Google and Apple app markets since 2011 and the developers have focused on making the app available to as many as possible. The original English PTSD Coach has also been evaluated in several studies. An open trial conducted in a VA setting suggested that users were very satisfied with the app and that they perceived it as helpful for their PTSD symptoms (Kuhn et al., 2014). To our knowledge, two randomized trials have investigated the efficacy of the PTSD Coach. Miner and colleagues (2016) investigated feasibility, acceptability and potential efficacy by comparing one month of access to the PTSD Coach to a wait-list condition. They reported that participants found the app moderately helpful and that they had learned to use the skills from the app to manage their symptoms, but the investigators found no statistically significant between-group effect size in terms of PTSD symptom improvement. Kuhn and colleagues (2017) also recruited trauma survivors from the community and compared three months of access to the PTSD Coach to a wait-list and found significant improvements in PTSD symptoms, depression and psychosocial functioning. Finally, in a small randomized study with primary care veterans, Possemato and colleagues (2016) compared access to the PTSD Coach with access to the app enhanced with guidance from a clinician and suggest that the addition of clinician support appears to increase the effects of self-management alone, but that a larger study is needed to confirm findings.

There has also been an international interest in the app. To our knowledge, work is ongoing in developing and evaluating versions in Danish, Norwegian, German and Dutch languages. Given that the PTSD Coach was developed in the US and within a military context, it is important to investigate if other language versions used in different clinical and cultural contexts also are valid and feasible tools. The purpose of this study was to preliminarily evaluate the user satisfaction, the perceived helpfulness and potential reductions in symptoms of posttraumatic stress (PTSS) and depression among participants using the Swedish version of the PTSD Coach.

## 2. Method

### 2.1. Design

This study was an uncontrolled pre-test post-test open trial in which all participants had access to the PTSD Coach app for four weeks.

### 2.2. Participants

Inclusion criteria were: age 18 years or above, Swedish speaker, access to a smartphone connected to the Google Play Store or the Apple App Store and experience of a potentially traumatic event, as defined by the DSM-5 (American Psychiatric Association, 2013), during the past five years. Exclusion criteria were ongoing severe psychiatric problems such as psychotic symptoms, severe depression, mania, ongoing substance abuse and risk for suicide.

### 2.3. Measures

#### 2.3.1. MINI International Neuropsychiatric Interview 6.0 (MINI 6.0)

The MINI 6.0 (Lecrubier et al., 1997) is a structured interview developed to screen for psychiatric disorders according to DSM-IV criteria. The interview covers the most common mood disorders, psychotic symptoms, substance abuse, anxiety disorders and suicidality and has demonstrated good inter-rater reliability All modules in the interview were used except the module assessing PTSD.

#### 2.3.2. Clinician-Administered PTSD Scale for DSM-5 (CAPS-5)

The CAPS-5 (Weathers et al., 2017) is a structured interview to assess PTSD according to DSM-5 criteria. In the current study partial PTSD was also assessed, requiring endorsement of one symptom from each of the B, C, D and E criteria.

Initial evaluations indicate that the interview shows strong inter-rater and test-retest reliability, as well as correspondence with earlier versions of the CAPS (Weathers et al., 2017). The Swedish version of the CAPS-5 was developed at the National Centre for Disaster Psychiatry, Uppsala University, and preliminary evaluations indicate similar properties as compared to the original version (unpublished data).

#### 2.3.3. PTSD Checklist-5 (PCL-5)

The PCL-5 (Blevins, Weathers, Davis, Witte, & Domino, 2015) is a self-report scale that assesses symptoms of PTSD and comprises 20 items corresponding to the DSM-5 PTSD symptom criteria. The respondents rate the intensity of each symptom during the past month using a 5-point scale from 0 ‘not at all’ to 4 ‘extremely’. Initial evaluations of the instrument indicate that it has good test-retest reliability, convergent and discriminant validity (Blevins et al., 2015). The Swedish version was developed using a standard back-translation process and show satisfactory psychometric properties (Sveen, Bondjers, & Willebrand, 2016).

#### 2.3.4. Patient Health Questionnaire-9 (PHQ-9)

The PHQ-9 (Kroenke, Spitzer, & Williams, 2001) assesses symptoms of depression and includes nine items. Respondents rate how much each symptom has been bothering them during the last two weeks using a 4-point scale from 0 ‘not at all’ to 3 ‘almost every day’. The instrument has shown good psychometric properties and adequate validity as a measure of symptoms of depression (Gilbody, Richards, Brealey, & Hewitt, 2007; Kroenke et al., 2001).

#### 2.3.5. PTSD Coach Survey

This instrument was developed by the original developers and investigators of the PTSD Coach app (Kuhn et al., 2014). The survey comprises 14 items that assess perceived helpfulness of the app and one item asking about user satisfaction. The original English version was translated to Swedish by the authors. Items are rated on a 5-point scale ranging from 0 ‘not at all’ to 4 ‘extremely’. The instrument was administered via the Internet after the participants gained access to the app. The 14 items measuring perceived helpfulness of the app demonstrated high internal consistency (Cronbach’s alpha = 0.96).

#### 2.3.6. Interview

A semi-structured interview via telephone was conducted after access to the app in order to get a more in-depth assessment of user patterns and experiences from interacting with the app. Examples of questions included were: ‘How have you used the app?’, ‘Which parts or functions of the app did you find most helpful?’ and ‘Do you have suggestions for improvement of the app?’ The answers were probed with follow-up questions.

### 2.4. PTSD Coach app

The source code for both IOS and Android versions was made available to the authors from the original developers (Kuhn et al., 2014) with the purpose of developing a Swedish version of the app. The content of the original app was translated from English to Swedish, new audio files were recorded and support information was adapted to match the available resources in Sweden. The material was then compiled into a Swedish version.

The app contains four main sections: *1. Learn*. This section provides psychoeducation about PTSD, e.g. prevalence, symptoms and how it develops, and information about treatment options; *2. Self-assessment*. This section includes the PTSD-Checklist Civilian version, a well-validated measure of symptoms of PTSD based on the DSM-IV. The respondent is given direct feedback on the severity of their symptoms and how it compares to their previous rating. Results are also presented graphically which enables the user to track their development of symptoms over time. Users can also schedule new assessments at different intervals and also schedule reminders; *3. Manage symptoms*. The section provides the user with different coping strategies addressing symptoms of PTSD. Examples of coping strategies include relaxation techniques, positive and adaptive self-statements and pleasant-events scheduling. Before each tool is presented the user is asked to provide a Subjective Unit of Distress Scale (SUDS) rating from 0 to 10 of their current distress. The SUDS rating is presented as a thermometer. This is repeated after the tool has been used and the user is provided with feedback on how their distress level changed. If the level is unchanged or has increased, users are prompted to try another tool; *4. Find support*. This section provides the user with contact details to different support functions, e.g. the national telephone number for emergency situations and different helplines that can be accessed via the telephone or the web. The users can also select contacts from their phone’s contacts app and add contact details to people of their own choice.

During this study, the Swedish version of the PTSD Coach was not publicly available on the Google Play Store or the Apple App Store. Instead, the participants accessed and downloaded the app via closed beta-testing functions, which made it possible to provide access as necessary.

### 2.5. Procedure

Participants were recruited from the community via advertisement in local newspapers and online. Interested individuals were instructed to e-mail their name and telephone number to a study coordinator. Individuals participating in an ongoing longitudinal observational study including adults with experiences of traumatic events were also asked for interest in participating in the current study. Interested individuals were assessed for eligibility via telephone. The participants who met inclusion criteria were assessed with the MINI and the CAPS-5. Before participants were provided access to the app, they were assessed via the Internet with the demographic questionnaire, the PCL-5 and PHQ-9. After completing the assessment, the participants accessed the app using their own devices via closed beta-testing functions on the Google Play Store and Apple App Store. After four weeks of access to the app, the participants were again assessed via the Internet with the PCL-5, PHQ-9 and the PTSD Coach Survey. Participants were interviewed via telephone about their experiences of using the app. The procedure was approved by the regional ethical vetting board in Uppsala (Reg. no.: 2015/258). All participants provided written informed consent.

During the project, technical difficulties made it necessary to modify the source code and recompile the code along with the content in Swedish several times in order to produce an acceptable working version. These difficulties caused delays in the project; participants who had been screened and completed the MINI and the CAPS interview had to wait several months before eventually accessing the app. However, the self-reported assessments via the Internet (PCL-5 and PHQ-9) were administered just before access to the app and after the four weeks of access.

### 2.6. Data analyses

Descriptive statistics were used to summarize the findings. The Wilcoxon paired samples test (2-tailed) was used to test for differences in PTSS and depression between the two assessments. The effect size Cohen’s *d*, corrected for the correlation between paired samples (Morris & DeShon, 2002), was calculated as a metric of the standardized mean difference between the pre- and the post-assessments. Two participants had missing data on one item each and these two values were imputed with the participants mean score on the observed items. All quantitative analyses were performed with the statistical package IBM® SPSS® Statistical package version 23.

Interviews were conducted via the telephone except for one participant who was interviewed in person. The interviews were conducted by one of the authors (MC) and the participants’ responses to the semi-structured interview were noted during the interviews. Data was analysed according to principles from qualitative description (Sandelowski, 2010). Notes were summarized in key units, and these key units were organized according to their common content (by MC). From this organization of key units, categories were identified (by MC). Another author (JS) reviewed the summaries and categories, and inconsistencies were resolved by discussion.

## 3. Results

### 3.1. Participant characteristics and type of trauma

**Figure 1.**
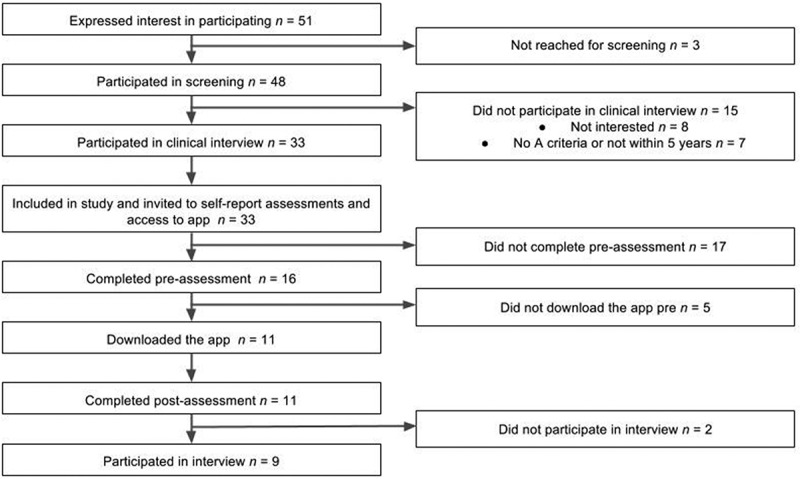
Participant flow through the study.

See Figure 1 for participant flow through the study. Of the 16 participants who participated in the pre-assessment, 13 were recruited via advertisement and three were recruited via the ongoing observational study. Eleven participants who completed both the pre- and post-assessment were included in the analyses. There was no difference in age between completers and non-completers, however non-completers had significantly lower scores on the PCL-5 (mean difference = 24.3, *p* < .05) and on the PHQ-9 (mean difference = 5.9, *p* < .05). Among completers, five participants met criteria for full PTSD and four met criteria for partial PTSD. Among non-completers, one participants met criteria for full PTSD and the remaining four did not meet criteria for partial PTSD. In the included sample the mean age was 38.6 years (range 23–55 years). Participant characteristics are shown in Table 1. Three participants reported that they had experienced an interpersonal trauma as their index event in the CAPS-5 and eight participants reported a non-interpersonal trauma as their index event.

**Table 1.** Participant characteristics (*n* = 11).

Characteristic	*n*
Gender	
Female	8
Male	3
Marital status	
Married/cohabiting	7
Single	4
Level of education (*n *= 10)	
Secondary school	1
Uncompleted university degree	3
Completed university degree	6
Employment	
Employed full time	5
Employed part time	2
Student	2
On sick leave	2
Unemployed	0
Treatment experience	
Psychotropic medication before traumatic event	3
Psychosocial treatment before traumatic event	6
Currently on psychotropic medication	1
Psychosocial treatment for traumatic event	7
Currently in psychosocial treatment/support	1

**Table 2.** Psychiatric morbidity as assessed with CAPS-5 and MINI 6.0 (*n* = 11).

Current psychiatric diagnosis	*n*
PTSD	5
Partial PTSD	4
Major depressive episode	2
Manic or hypomanic episode	-
General anxiety disorder	4
Panic disorder	2
Agoraphobia	3
Social phobia	4
Obsessive compulsive disorder	5
Any substance-related disorder	-
Any psychotic disorder	-
Anorexia nervosa	-
Bulimia nervosa	1
Meets criteria for more than 1 disorder^a^	8
No disorder	2

^a^Includes meeting criteria for either full or partial PTSD.

**Table 3.** PCL-5 and PHQ-9 scoring distribution at pre- and post-assessment (*n *= 11).

	Pre-assessment		Post-assessment	
	*M* (*SD*)	Range	*M* (*SD*)	Range
PCL-5 Total	36.89 (14.33)	23–65	31.68 (15.91)	10–62
Intrusion	9.18 (4.64)	2–17	7.81 (4.11)	3–16
Avoidance	4.10 (2.43)	0–8	4.18 (2.63)	0–8
Cognition and mood	13.34 (6.20)	7–25	11.82 (6.91)	3–23
Arousal and reactivity	10.27 (3.52)	6–17	7.86 (4.97)	1–17
PHQ-9 Total	10.91 (6.24)	4–24	9.18 (6.43)	3–23

PCL-5 = PTSD Checklist 5, PHQ-9 = Patient Health Questionnaire 9.

**Table 4.** PTSD Coach Survey scores (*n* = 11).

	Item	*M* (*SD*)
1.	Helping me learn about symptoms of PTSD	1.55 (1.21)
2.	Helping me learn about treatments for PTSD	1.45 (1.29)
3.	Helping me find effective ways of managing my symptoms	1.45 (1.21)
4.	Helping me feel more comfortable in seeking support	1.64 (1.12)
5.	Helping me feel there is something I can do about my PTSD	1.91 (1.38)
6.	Helping me track my symptoms	1.09 (1.22)
7.	Helping me know when I’m doing better or when I’m doing worse	1.18 (1.08)
8.	Increasing my access to additional resources	1.36 (1.36)
9.	Providing practical solutions to the problems I experience	1.55 (1.37)
10.	Helping me overcome the stigma of seeking mental health services	1.27 (1.27)
11.	Helping me better understand what I have been experiencing	1.36 (1.50)
12.	Enhancing my knowledge of PTSD	1.82 (1.40)
13.	Helping clarify some of the myths about PTSD	1.00 (1.34)
14.	Providing a way for me to talk about what I have been experiencing	0.91 (0.94)
15.	Overall how satisfied are you with the PTSD Coach?	1.55 (1.21)

Helpfulness and satisfaction ratings: 0 = not at all; 1 = slightly; 2 = moderately; 3 = very; 4 = extremely.

### 3.2. Psychiatric morbidity

According to the CAPS-5 interview, five individuals met criteria for full PTSD and four met the criteria for partial PTSD at the time of the interview. Partial PTSD was defined as meeting one or more criteria in each of the three symptom groups, i.e. B, C and D (Breslau, Lucia, & Davis, 2004). According to the MINI 6.0, two participants met criteria for one psychiatric diagnosis and eight participants met criteria for more than one diagnosis (Table 2).

### 3.3. PCL-5 and PHQ-9

The scores on the PCL-5 decreased from pre- to post-assessment with a moderate effect size (mean difference = 5.2; 95% CI: −1.7, 12.1; Cohen’s *d* = 0.51), however, this change was not statistically significant (*Z *= − 1.42, *p* = .16). The PHQ-9 scores also decreased with a moderate effect size (mean difference = 1.7; 95% CI: −0.3, 3.7; Cohen’s *d* = 0.58), however, this also failed to reach statistical significance (*Z *= − 1.85, *p *= .06) (see Table 3). 

### 3.4. PTSD Coach Survey

The item ratings on the PTSD Coach Survey ranged between 0.91 and 1.91, indicating that the participants were, in general, slightly to moderately satisfied with the app and found it slightly to moderately helpful (see Table 4).

### 3.5. Interviews

Nine participants were interviewed by telephone shortly after completing the post-assessment with semi-structured questions and one participant was interviewed in person. The data from the interviews were summarized in the following descriptive categories.

#### 3.5.1. Helpful components

Exercises such as breathing and relaxation techniques were highlighted by participants as helpful (*n =* 4). Furthermore, reading and learning more about PTSD was also perceived as helpful (*n =* 3). One participant described that the information provided in app had a normalizing effect on her experiences. Also, the advantage of having the information always available and being able to go back at any time was perceived as helpful (*n =* 1). The RID-tool (Relax, Identify, Decide; *n =* 3) and the SUDS rating before and after an exercise (*n =* 2) were also put forth as helpful. One participant valued the option to schedule reminders for planned activities and exercises.

#### 3.5.2. Changed coping strategies

Some participants felt that the app had helped them to reflect on their situation and identify difficulties (*n =* 3) and to understand their difficulties in a better way (*n =* 1). Participants also reported that knowing that they had the app had given a sense of support and a sense of feeling calmer (*n =* 2).

#### 3.5.3. Using the app when alone

Some of the participants preferred to use the app at home (*n =* 3) and in quiet situations (*n =* 2). For example, one participant used it when having trouble falling asleep and another used it around bedtime.

#### 3.5.4. Less usage than intended

Several individuals said that they had not used the app as much as they had intended to (*n =* 5) and some of the reasons stated were that it did not meet expectations (*n =* 1), eye problems (*n =* 1) and difficult app structure (*n =* 1).

#### 3.5.5. Technical problems

Two participants experienced technical problems, in which the playback of an audio track during an exercise ended prematurely or did not start properly.

#### 3.5.6. Suggestions to improve the app

Some participants suggested that the app could benefit from more structure (*n =* 4), that it would make it easier to follow, and suggested more personalized settings (*n =* 3), such as being able to change colour and voices in the app (*n =* 1) or create shortcuts from the homescreen to specific content in the app (*n =* 1). Two participants stated that the app was difficult to figure out and that it could benefit from a more explicit overall purpose and additional guidance throughout the different parts of the app. One participant stated that they had not understood the purpose of some of the content and suggested improved rationales for some of the exercises. Another participant suggested that the app could be used in combination with other services rather than as a stand-alone intervention.

## 4. Discussion

The overall purpose of this study was to evaluate the user satisfaction, perceived helpfulness and potential reductions of PTSS and symptoms of depression when using the Swedish version of the PTSD Coach. To this end, a Swedish version of the PTSD Coach was developed based on the source code from the original English language version. Participants from the community were recruited and invited to use the app. The participants were in general slightly to moderately satisfied with the app and found it to be slightly to moderately helpful. In addition, nominal reductions in PTSS and symptoms of depression with moderate effect sizes were observed, although these failed to reach statistical significance. According to interviews, the participants found specific functions of the app helpful such as relaxation exercises, the RID-tool and the SUDS rating. They also had suggestions for improvements such as a clearer structure and linearity in the app, and being given more possibilities of personalizing the app. Several participants indicated that they had not used the app as much as they intended to, or thought that they would be, when signing up for the study.

The current results both departs and coheres with previous investigations of other language versions of the app. In their preliminary investigation, Kuhn and colleagues (2014) generally found higher ratings on the PTSD Coach Survey, rating it as moderately to very helpful or being moderately to very satisfied with the app. One reason for the lower ratings in the present study may be that the participants in the current investigation were recruited from the community whereas the participants in the Kuhn et al. (2014) study were attendees at a VA residential treatment programme. Thus, participants in the Kuhn et al. (2014) study may have had increased contact with the research staff, and hence were more positive to the content of the app. In addition, in the current project, several technical difficulties were encountered in the process of producing the Swedish app. The resulting delay between recruitment and access to the app may have led to participants having a more negative attitude towards the app, which, in turn, may have contributed to the lower helpfulness and usability scores. It is important that research aiming to evaluate technical solutions such as mobile apps find and maintain reliable technical competencies required for successful implementation of the technology. Also, several of the participants indicated that they had not used the app as much as they thought they would have. Although the usage data were based on self-report, this observation is in line with a previous investigation of the use of the original English version of the app. Owen and colleagues (2015) analysed user data from their apps accessed on the Apple App Store and Google Play Store and found that 61% of users who downloaded the app returned to the app the next day, and that 52% of users continued to use the app or used it at least one time beyond the first week of download. This indicates that it is important that mobile apps targeting mental health problems are designed so that they engage users in accessing the app content and that use is maintained over time. One such example would be providing tutorials or examples for how to use the app. Another method to increase usage and adherence to key components could be to combine access to the app with therapist support via e-mail, phone or instant messaging as suggested by the Supportive Accountability model (Mohr, Cuijpers, & Lehman, 2011).

Despite these difficulties, there was a nominal reduction in PTSS with a moderate effect size, albeit not reaching statistical significance, which is in line with a recent investigation (Miner et al., 2016). Using a controlled wait-list design with a sample recruited from the community, Miner and colleagues (2016) found a non-significant between-group effect size of 0.25 after one month of access to the app. However, they found a moderate within-group effect size of 0.59, indicating a reduction in PTSS of a magnitude similar to the reductions found in the current study. A more recent investigation increased the time of access to the app to three months of use and found a larger between-group effect size (Kuhn et al., 2017). Future studies may therefore benefit from extending the trial period beyond one month of access to the app, as used in the current study, as it might be a too short period of time to produce marked reductions in symptoms. Future studies of the PTSD Coach and other similar apps may also focus on investigating whether usage of certain features in the apps are related to specific reductions in distress. Combining rigorous trial designs where app-usage data is continuously collected (logins, duration of usage, duration at certain elements, etc.) with, for example, ecological momentary assessment (Shiffman, Stone, & Hufford, 2008), in which data on symptoms/distress are collected with high frequency, could yield more detailed information about the potential mechanisms of change related to app usage.

The current investigation has several limitations. First, the project was hindered by technical difficulties which caused delays in participants accessing the app, and this may have had a negative influence on the attitude of the participants towards the app. Second, the study was small with participants recruited from the community via advertisement and from an ongoing study, which may negatively affect the representativeness of the included sample. The small sample size also limits the power to adequately detect change over time in terms of the symptoms assessed. In addition, the study lacks a control group so we do not know whether the changes in PTSS and depression were due to access to the app or merely a result of time. In the current study, negative effects were not formally assessed. Negative effects such as deterioration, adverse events, novel symptoms and non-response are understudied in psychological/psychosocial intervention research (Jonsson, Alaie, Parling, & Arnberg, 2014). In future trials, we intend to also assess any negative effects associated with using the Swedish version of the PTSD Coach. Finally, it may be argued that participation in a beta-testing programme leads to the overall user experience not mimicking real-world usage and may thus negatively influence the ecological validity and generalizability of the findings.

To conclude, the current investigation suggest that the Swedish version of the PTSD Coach was perceived as slightly to moderately helpful and that participants were slightly to moderately satisfied with the app. Furthermore, participants experienced substantive but not statistically significant reductions in PTSS and depression during access to the app. Participants also indicated that they had not used the app as much as intended, and had suggestions for improvements. As such, it is a first step towards investigating the efficacy of the PTSD Coach in a Swedish context. As a next step, the experiences gained in the current study will be used in modifying and refining the Swedish version of the PTSD Coach. Modifications will include using the updated code from the developers including a refreshed user interface and clarification of some of the rationales for the different exercises. Examples of different use cases will also be included to provide guidance when using the app. This new version will then be subjected to evaluation in a more rigorous study using a controlled design.

## References

[CIT0001] American Psychiatric Association (2013). . Arlington, VA: American Psychiatric Association.

[CIT0002] Anderson R. (2008, 10 22). New MRC guidance on evaluating complex interventions. , 337(1), a1937–9. 10.1136/bmj.a1937 18945728

[CIT0003] Andersson G., & Titov N. (2014). Advantages and limitations of internet-based interventions for common mental disorders. , 13(1), 4–11. 10.1002/wps.20083 PMC3918007 24497236

[CIT0004] Arnberg F. K., Bergh Johannesson K., & Michel P.-O. (2013). Prevalence and duration of PTSD in survivors 6 years after a natural disaster. , 27(3), 347–352. 10.1016/j.janxdis.2013.03.011 23660149

[CIT0005] Arnberg F. K., Gudmundsdóttir R., Butwicka A., Fang F., Lichtenstein P., Hultman C. M., & Valdimarsdóttir U. A. (2015). Psychiatric disorders and suicide attempts in Swedish survivors of the 2004 southeast Asia tsunami: A 5 year matched cohort study. , 2(9), 817–824. 10.1016/S2215-0366(15)00124-8 26236006

[CIT0006] Arnberg F. K., Linton S. J., Hultcrantz M., Heintz E., & Jonsson U. (2014). Internet-delivered psychological treatments for mood and anxiety disorders: A systematic review of their efficacy, safety, and cost-effectiveness. , 9(5), e98118. 10.1371/journal.pone.0098118 PMC4028301 24844847

[CIT0007] Blevins C. A., Weathers F. W., Davis M. T., Witte T. K., & Domino J. L. (2015). The posttraumatic stress disorder checklist for DSM-5 (PCL-5): Development and initial psychometric evaluation. , 28(6), 489–498. 10.1002/jts.22059 26606250

[CIT0008] Breslau N., Lucia V. C., & Davis G. C. (2004). Partial PTSD versus full PTSD: An empirical examination of associated impairment. , 34(7), 1205–1214. 10.1017/s0033291704002594 15697047

[CIT0009] Frans O., Rimmö P.-A., Aberg L., & Fredrikson M. (2005). Trauma exposure and post-traumatic stress disorder in the general population. , 111(4), 291–299. 10.1111/j.1600-0447.2004.00463.x 15740465

[CIT0010] Gilbody S., Richards D., Brealey S., & Hewitt C. (2007). Screening for depression in medical settings with the Patient Health Questionnaire (PHQ): A diagnostic meta-analysis. , 22(11), 1596–1602. 10.1007/s11606-007-0333-y PMC2219806 17874169

[CIT0011] Goldstein R. B., Smith S. M., Chou S. P., Saha T. D., Jung J., Zhang H., … Grant B. F. (2016). The epidemiology of DSM-5 posttraumatic stress disorder in the USA: Results from the national epidemiologic survey on alcohol and related conditions-III. , 51(8), 1137–1148. 10.1007/s00127-016-1208-5 PMC4980174 27106853

[CIT0012] Jonsson U., Alaie I., Parling T., & Arnberg F. K. (2014). Reporting of harms in randomized controlled trials of psychological interventions for mental and behavioral disorders: A review of current practice. , 38(1), 1–8. 10.1016/j.cct.2014.02.005 24607768

[CIT0013] Kroenke K., Spitzer R. L., & Williams J. B. (2001). The PHQ-9: Validity of a brief depression severity measure. , 16(9), 606–613. 10.1046/j.1525-1497.2001.016009606.x PMC1495268 11556941

[CIT0014] Kuhn E., Greene C., Hoffman J., Nguyen T., Wald L., Schmidt J., … Ruzek J. (2014). Preliminary evaluation of PTSD Coach, a smartphone app for post-traumatic stress symptoms. , 179(1), 12–18. 10.7205/MILMED-D-13-00271 24402979

[CIT0015] Kuhn E., Kanuri N., Hoffman J. E., Garvert D. W., Ruzek J. I., & Taylor C. B. (2017). A randomized controlled trial of a smartphone app for posttraumatic stress disorder symptoms. , 85(3), 267–273. 10.1037/ccp0000163 28221061

[CIT0016] Lecrubier Y., Sheehan D. V., Weiller E., Amorim P., Bonora I., Harnett Sheehan K., … Dunbar G. C. (1997). The Mini International Neuropsychiatric Interview (MINI). A short diagnostic structured interview: Reliability and validity according to the CIDI. , 12(5), 224–231.

[CIT0017] Mandelli L., Petrelli C., & Serretti A. (2015). The role of specific early trauma in adult depression: A meta-analysis of published literature. Childhood trauma and adult depression. , 30(6), 665–680. 10.1016/j.eurpsy.2015.04.007 26078093

[CIT0018] Miner A., Kuhn E., Hoffman J. E., Owen J. E., Ruzek J. I., & Taylor C. B. (2016). Feasibility, acceptability, and potential efficacy of the PTSD Coach app: A pilot randomized controlled trial with community trauma survivors. , 8(3), 384–392. 10.1037/tra0000092 27046668

[CIT0019] Mohr D. C., Cuijpers P., & Lehman K. (2011). Supportive accountability: A model for providing human support to enhance adherence to eHealth interventions. , 13(1), e30. 10.2196/jmir.1602 PMC3221353 21393123

[CIT0020] Morris S. B., & DeShon R. P. (2002). Combining effect size estimates in meta-analysis with repeated measures and independent-groups designs. , 7(1), 105–125. 10.1037/1082-989x.7.1.105 11928886

[CIT0021] Olff M. (2015). Mobile mental health: A challenging research agenda. , 6, 27882. 10.3402/ejpt.v6.27882 PMC4439418 25994025

[CIT0022] Owen J. E., Jaworski B. K., Kuhn E., Makin-Byrd K. N., Ramsey K. M., & Hoffman J. E. (2015). mHealth in the wild: Using novel data to examine the reach, use, and impact of PTSD Coach. , 2(1), e7. 10.2196/mental.3935 PMC4607374 26543913

[CIT0023] Possemato K., Kuhn E., Johnson E., Hoffman J. E., Owen J. E., Kanuri N., … Brooks E. (2016). Using PTSD Coach in primary care with and without clinician support: A pilot randomized controlled trial. , 38, 94–98. 10.1016/j.genhosppsych.2015.09.005 26589765

[CIT0024] Sandelowski M. (2010). What's in a name? Qualitative description revisited. , 33(1), 77-84. 10.1002/nur.20362 20014004

[CIT0025] Shiffman S., Stone A. A., & Hufford M. R. (2008). Ecological momentary assessment. , 4, 1–32. 10.1146/annurev.clinpsy.3.022806.091415 18509902

[CIT0026] Sveen J., Bondjers K., & Willebrand M. (2016). Psychometric properties of the PTSD checklist for DSM-5: A pilot study. , 7, 30165. 10.3402/ejpt.v7.30165 PMC4838990 27098450

[CIT0027] The Internet Foundation in Sweden (2017). *Swedes and the internet 2017* . Stockholm, Sweden: Author Retrieved from http://www.soi2017.se/

[CIT0028] Weathers F. W., Bovin M. J., Lee D. J., Sloan D. M., Schnurr P. P., Kaloupek D. G., … Marx B. P. (2017). The clinician-administered PTSD scale for DSM-5 (CAPS-5): Development and initial psychometric evaluation in military veterans. . doi:10.1037/pas0000486 PMC5805662 28493729

[CIT0029] Witteveen A. B., Bisson J. I., Ajdukovic D., Arnberg F. K., Johannesson K. B., Bolding H. B., … Nordanger D. O. (2012). Post-disaster psychosocial services across Europe: The TENTS project. , 75(9), 1708–1714. 10.1016/j.socscimed.2012.06.017 22835920

